# Effects of Different Types of Exercise Training on Fine Motor Skills and Testosterone Concentration in Adolescents: A Cluster Randomized Controlled Trial

**DOI:** 10.3390/ijerph18168243

**Published:** 2021-08-04

**Authors:** Justė Knatauskaitė, Kazimieras Pukėnas, Laima Trinkūnienė, Henning Budde

**Affiliations:** 1 Institute of Sport Science and Innovations, Lithuanian Sports University, 44221 Kaunas, Lithuania; henning.budde@medicalschool-hamburg.de; 2 Department of Health Promotion and Rehabilitation, Lithuanian Sports University, 44221 Kaunas, Lithuania; kazimieras.pukenas@lsu.lt; 3 Department of Physical and Social Education, Lithuanian Sports University, 44221 Kaunas, Lithuania; laima.trinkuniene@lsu.lt; 4 Institute for Systems Medicine, Faculty of Human Sciences, MSH Medical School Hamburg, 20457 Hamburg, Germany

**Keywords:** school, cardiovascular exercise, coordinative exercise, fine motor skills, testosterone

## Abstract

We sought to compare the effect of two types of exercise training on fine motor skills and to establish their association with the salivary testosterone. Research participants, 135 adolescents (68 adolescent females; mean age = 12.76, SD = 0.85) were assigned into three groups: coordinative exercise (CE), cardiovascular exercise (CVE), and the control group (CON). Fine motor skills were assessed with a drawing trail test and salivary testosterone concentration was measured before and after 10 weeks of intervention. There were no significant changes in fine motor skills and testosterone concentration after either CE or CVE training. However, a significant positive correlation between post-test fine motor skills and post-test testosterone concentration was found after the CVE training. One type of exercise training cannot be singled out as more effective for fine motor skills and testosterone activity. Nevertheless, our results showed a relationship between fine motor skills and testosterone concentration after the CVE training. Thus, the type of exercise training is important in the exercise-induced testosterone effect on fine motor skills.

## 1. Introduction

Regular physical activity (PA) is of paramount importance for children and adolescents. Positive PA effects include benefits to mental and physical development as well as overall health status [1,2,3]. Motor performance relates to cognitive performance [4], which further influences an academic achievement [5]. For example, Koutsandréou et al. [6] showed better working memory after either cardiovascular or motor exercise training in children, with the latter having a greater effect. Moreover, Grissmer et al. [7] reported fine motor skills as predictor of academic achievement. The studies mostly analyzed the fine motor skills in children [8,9,10]. Hence, the literature about adolescents is scarce.

Fine motor skills require a coordinated movement of hand, wrist, and fingers [10]. The movements are small and delicate. These abilities are important in daily and learning activities, for example, eating, writing [11] and require accuracy, fluency, and attention [10]. The intervention improving fine motor skills is of particular interest at school where the priority is cognitive performance and academic achievement [12,13]. However, there is a lack of evidence regarding the effect of chronic exercises, which can be called exercise training [14], on fine motor skills in adolescents.

The type of exercise is considered to be one of the exercise factors influencing exercise outcomes [15]. Cardiovascular exercise (CVE) training induces increased blood flow in the motor cortex and thus influences changes in brain areas responsible for motor control [16]. While CVEs comprise automatic simple movements using large muscle groups with low cognitive demands [17,18], increasing attention is now being paid to coordinative exercise (CE) training as one of the motor training interventions beneficial in the improvement of cognitive skills [6]. Probably because CE requires coordinative abilities and cognition, this exercise type has been found to engage the cerebellum [19,20], which is responsible for fine motor skills development and control [4]. This exercise type has been indicated as less metabolic and mainly related to motor learning [21]. While both CVE and CE training impact brain functioning [18], the mechanisms underlying these effects are different [18]. For example, an animal study done by Black, Isaacs, Anderson, Alcantara, and Greenough [22] reported that motor learning generated new synapses in cerebellar cortex while angiogenesis was caused by cardiovascular endurance exercises. The primary aim of this study was to analyze the benefits of CVE and CE training on fine motor skills in an adolescent population. We expected that CE training would have a higher impact on fine motor skills than CVE training.

A possible moderator in the exercise effect on fine motor skills is testosterone [23]. Studies with adults showed an increased testosterone concentration after exercise training [24,25]. Results of preadolescent children, reported by a recently published study [26], revealed no change in testosterone concentration after motor vs. CVE training. However, Niemann et al. [27] concluded that the testosterone response to acute exercise was influenced by sustained PA in habitually low-active preadolescent children, but with no clear indication of the type of exercise training. Furthermore, the neuroprotective effect of testosterone influences various neural levels which are responsible for motor functions (e.g., motor neurons) [28]. It is well established that motor neurons maintain the androgen receptors and thus testosterone is involved in motor functions [29,30]. Wegner et al. [23] demonstrated an association between testosterone and fine motor skills among high school students independent of the acute stressor (psychosocial vs. CVE). Thus, the second aim of our study was to determine the testosterone concentration after the physical stress of CVE vs. CE training and establish whether testosterone concentration was related to fine motor skills among adolescents. We expected the relationship between changes in testosterone concentration and altered fine motor skills to be independent on the type of exercise training.

## 2. Materials and Methods

### 2.1. Participants

A cluster randomized controlled trial was implemented in six schools. A group of research participants from each of the schools (135 adolescents in total, 68 adolescent females, mean age = 12.76, SD = 0.85) was randomly assigned into one of the three groups: control group (CON) (*n* = 46; 23 adolescent females), coordinative exercise (CE) (*n* = 41; 22 adolescent females), and cardiovascular exercise (CVE) group (*n* = 48; 23 adolescent females) (Table 1). All participants gave their signed informed assent forms, and parents of all participants signed informed consent forms in accordance with the Declaration of Helsinki. The study protocol was approved by the Kaunas Regional Biomedical Research Ethics Committee (Lithuania). Participant inclusion criteria were the absence of obesity, physical (e.g., traumatic injury) and mental impairments (e.g., mental retardation), and no consumption of psychotropic medications. Additionally, the PA level was evaluated. All participants were moderately active according to Tammelin et al. [31]. It means that participants spent 2–3 hours/week in a moderate-to-vigorous PA outside school hours (e.g., a walk in the forest with the family, playing with peers in the yard). Adolescents were not involved in any professional training or other organized PA program. All participants had to attend no less than 75% of the exercise training sessions [6].

### 2.2. Procedure

Participants were familiarized and performed all necessary tests one week before the intervention (first assessment), including the shuttle run test, flamingo balance test, hand–eye coordination test, and drawing trail test. We also took their height, weight, and pubertal maturation measurements and recorded the values of salivary testosterone. All tests and the measurement of testosterone were repeated one week after the 10-week exercise intervention.

### 2.3. Measures

The participant’s height was measured to the nearest of 0.1 cm using the Harpenden anthropometer set (Holtain Ltd., Crymych, Dyfed, Wales, UK). The coefficient of reliability above 95% has been reported for the height measurement with Harpenden stadiometer [32].

The participant’s weight was measured with the body composition scale (TBF-300; Tanita, Manchester, UK). The high reliability (*R* = 0.991) and validity (*r* = 0.972) has been reported [33].

The participant’s pubertal maturation was assessed using the Tanner stages (I–V) by drawings of breasts, genitals, and pubic hair. It is a self-assessment tool that can be used in larger studies to assess pubertal status [34]. It was confirmed in the study carried out by Chavarro et al. [35]: validity of self-reported pubertal staging for adolescents varied from *r* = 0.50 to *r* = 0.99.

The heart rate was measured using the heart rate monitor (Polar FT1, Kempele, Finland). Goodie et al. [36] found significant within-subject correlations (*r* > 0.90) between the Polar heart rate monitor and electrocardiography. 

The shuttle run test evaluates cardiorespiratory fitness. On this test, the participant must run back and forth between two lines (20 m apart) with increasing intensity. The designated speed is 8 km/hour initially, and this speed increases by 0.5 km/hour every minute. When the participant cannot maintain the planned pace between the two lines, the test is stopped, and the level and number of shuttles run to that point are recorded. This test is a reliable and valid method to estimate maximal oxygen uptake in adolescents [37]. The score of the test has a moderate-to-high criterion-related validity for estimating maximal oxygen uptake (*r*_p_ = 0.78–0.95) [38].

The flamingo balance test is one of the Eurofit Fitness Testing Battery tests [39]. Participants maintain their balance on a beam (50 cm length, 4 cm height, and 3 cm width) for 1 min. Every attempt to maintain balance is counted and then recorded as a point. Small values (closer to 1) indicate better balance. The high reliability of the flamingo balance test has been reported (ICC = 0.910) [40].

The hand–eye coordination test [41] requires the participant to stand behind a line two meters away from a wall. The participant then throws a tennis ball with his/her dominant hand against the wall and catches it with the opposite hand. The ball is then thrown back against the wall and caught with the initial throwing hand. This cycle of throwing and catching is repeated for 30 seconds, and the number of successful catches is recorded. The intra-class correlation coefficient of 0.72 has been reported [42]. 

The three tests were used to prove the effectiveness of each exercise training program.

The drawing trail test [43] is a subtest from the Movement Assessment Battery for Children-2 (M-ABC-2) which assesses fine motor skills and can be implemented in a group of participants. The participant has to draw a line between two solid lines (1.5 mm apart) as accurately as possible and without lifting a pen. Each crossing the line or raising the pen is marked as a mistake. A higher score (sum of the mistakes) in the drawing trail test means more mistakes made. Only the preferred hand is tested. The good reliability has been reported for the M-ABC-2 (test–retest reliability of the total test score—0.80) [44], as cited in Henderson et al., 2007.

Salivary testosterone measurements are used as a reliable indicator in exercise sciences [45]. The significant relationship between plasma and salivary testosterone has been reported (*r* = 0.71–0.73) [45]. While seated, the participant must fill a tube (about ¾ of 2 mL) with saliva, using a straw for better filling. Pre-test and post-test samples were collected in the afternoon (2–3 p.m.) and stored with a cold pack for about two hours and then later stored at −20 °C. Hormone concentration within the saliva sample was measured using the enzyme-linked immunosorbent assay (IBL, Hamburg, Germany). The samples were not measured in duplicates, and the inter-assay coefficient of variation was 13.68%.

### 2.4. Intervention 

The exercise intervention period was 10 weeks and did not include holidays. Experimental groups had common physical education classes twice per week for 45 minutes each, and additional exercise sessions were held three times per week for 45 minutes after the class time. Exercise sessions were led by a Physical Education teacher who was instructed and provided with a material of exercises. Exercise plan was based on the Lithuanian book “Modern Physical Education Lesson: Games” [46] together with the prepared exercises of researchers (Table 2). The Physical Education teacher was able to modify the exercises according to participants’ skills. All training sessions took place in the gym. The teacher was blinded to the aim of experiment.

The intensity of the exercise program was evaluated by calculating the mean values of the heart rate during the exercise trainings. Three randomly selected adolescents in each school wore a heart rate monitor watch with a chest strap in every session. The following formula was used to calculate the maximum heart rate (HRmax) [47]: 208 − 0.7 × age. The intensity of exercise training in both interventional groups can be described as moderate (64–76% HRmax) [48].

The after-school CE training program was composed of tasks demanding motor skills. The exercises focused on coordination skills, body balance, trunk stability, spatial orientation (e.g., throwing a ball to the target, juggling with tennis balls while standing on the unstable platform). Jumping ropes, balls, rackets, benches, sports bows, and unstable platforms were used as the equipment to create an interesting and motivating environment for participants [6]. Motor tests were not included in the training sessions as exercises. 

The CVE training program consisted of exercises with little motor skill demands. The main goal was to focus on the improvement of cardiovascular endurance. Adolescents performed different cyclic exercises such as fast walking, jumping, and played games focused on aerobic endurance (e.g., catching games, a relay with a speed-demanding task). 

The CON participants underwent no additional after-school exercises beyond the Physical Education classes two times per week for 45 minutes that were common to all groups. During the time when other participants were undergoing CE or CVE training programs, CON participants were required to take part in assisted homework sessions in order to prevent any attention bias and to control for test–retest effects.

### 2.5. Data Analysis

Statistical procedures were conducted using the software SPSS 25.0 for Windows (IBM, New York, NY, USA). For statistical analysis, salivary testosterone values were log-transformed to achieve a normal distribution. We used a 2 (test times) × 3 (exercise conditions) repeated-measures analysis of variance (ANOVA) with time as the within-subject factor and groups as the between-trial factors. Analyses have been controlled for sex, age, BMI, and puberty (according to the Tanner stages) using an ANCOVA, where no significant effects were found. Thus, the original results were reported. Calculations for statistical power (observed power, *OP*) were performed and the partial *η*^2^ was estimated (≤0.039: no effect, 0.04–0.24: minimum, 0.25–0.63: moderate, ≥0.64: strong according to Ferguson [49]) as a measure of the experimental trial effect size.

The participants’ scores on the shuttle run, flamingo balance, hand–eye coordination, and drawing trail tests were not normally distributed (according to a one-sample Kolmogorov–Smirnov test (*p* < 0.050)) and the data were analyzed using nonparametric tests. The nonparametric tests were based on ranking values from low to high, and then examining the distribution of sum-of-ranks between groups or trials. The Wilcoxon signed ranks test was used to compare two related samples in each group. The Kruskal–Wallis H test was used to compare three independent samples. Two independent sample comparisons were conducted with a Mann–Whitney U test to compare the samples pairwise between the three groups. The following equation was used to determine the effect size *r* [50]: *r* = Z/√*n*; where Z—the z-score, and *n*—the total number of the samples; *r* = 0.10 is for small effect, *r* = 0.30—medium effect, and *r* = 0.50—large effect.

Spearman’s correlation coefficient was used as a measure of the relationship between the pre-, post-test, and difference (post-test–pre-test) values of salivary testosterone and fine motor skills with the effect size *r* (0.10 to 0.29 = a small relationship; 0.30 to 0.49 = a medium relationship; 0.50 to 1.00 = a large relationship according to Cohen [51]). 

## 3. Results

### 3.1. Cardiovascular Performance

Two-related sample analysis revealed a significant improvement of cardiorespiratory fitness (Table 3) from pre-test to post-test in the CON (*p* < 0.001, *r* = 0.71) and in the CVE group (*p* < 0.001, *r* = 0.83). The cardiovascular performance significantly decreased in the CE group after the intervention (*p* < 0.001, *r* = 0.70).

According to the Kruskal–Wallis H test, three groups significantly differed after the intervention (*p* < 0.001; before: *p* = 0.656). After the follow-up between-group analysis (Mann–Whitney U test), the participants’ cardiovascular performance significantly differed between the two experimental groups (CVE and CE) after the intervention (*p* = 0.001, *r* = 0.55). Additionally, the cardiovascular performance after the intervention significantly differed between the CON and CE group—values in the CON were higher (*p* < 0.001, *r* = 0.47). The difference between the cardiovascular performance of the CON and CVE group was not significant after the intervention (*p* = 0.288, *r* = 0.12).

### 3.2. Static Balance Performance

The flamingo balance test was used to evaluate static balance performance (Table 3). Participants in the CE group improved their static balance after the intervention (*p* = 0.001, *r* = 0.53). However, participants in the CVE group and CON showed a diminished static balance performance after the intervention (CVE: *p* = 0.082, *r* = 0.25; CON: *p* = 0.001, *r* = 0.50). 

After conducting the Kruskal–Wallis H test, all groups significantly differed after the intervention (*p* = 0.002; before: *p* = 0.399). The results in the CE group were significantly better compared with CVE and CON groups (*p* = 0.003, *r* = 0.31; *p* = 0.001, *r* = 0.34, respectively). The static balance performance after the intervention did not differ between the CVE and CON groups (*p* = 0.479, *r* = 0.07).

### 3.3. Hand–Eye Coordination Performance

The second test for gross motor skills was a hand–eye coordination test (Table 3). These results were significantly better in both experimental groups after the intervention (CE: *p* < 0.001, *r* = 0.74; CVE: *p* < 0.001, *r* = 0.76). The participants’ hand–eye coordination performance decreased in the CON, but it was not statistically significant (*p* = 0.473, *r* = 0.12).

The between-group analysis revealed that the three groups significantly differed after the intervention (*p* < 0.001; before: *p* = 0.231). Hand–eye coordination performance was significantly better in the CE group comparing to the CON (*p* < 0.001, *r* = 0.51) after the intervention. Moreover, the results of the hand–eye coordination test were higher in the CVE group comparing to the CON (*p* < 0.001, *r* = 0.48). The results of the two experimental groups were not significantly different after the intervention (*p* = 0.757, *r* = 0.04).

### 3.4. Fine Motor Skills

Fine motor skills were evaluated using the drawing trail test (Table 3). There were no significant changes in fine motor skills in both the CVE and CE groups after the intervention, although the participants in the CVE group made fewer mistakes (CVE: *p* = 0.791, *r* = 0.04; CE: *p* = 0.332, *r* = 0.15). The results of the CON significantly worsened after 10 weeks of the intervention (*p* = 0.039, *r* = 0.30). 

After comparing the values of the drawing trail test between the three groups, no significant differences were found either before (*p* = 0.716) or after (*p* = 0.380) the intervention. 

### 3.5. Testosterone Concentration

**Figure 1:**
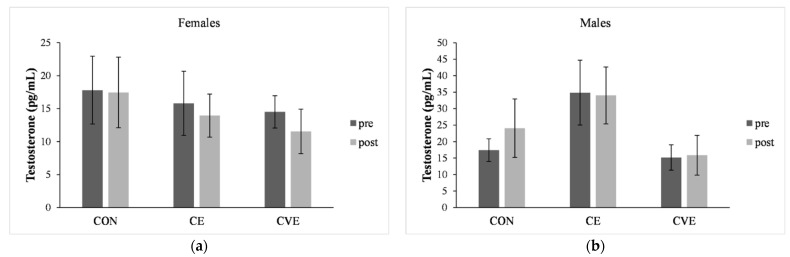
Pre- and post-test means of testosterone concentration in females (**a**) and males (**b**) are shown for the control group (CON), the coordinative exercise (CE), and the cardiovascular exercise (CVE) group. Error bars represent 95% confidence intervals for the mean.

A one-way ANOVA showed a different testosterone concentration between females and males before (*F*_1_ = 6.18, *p* = 0.014, *η*^2^ = 0.05, *OP* = 0.69) and after the intervention (*F*_1_ = 11.89, *p* = 0.001, *η*^2^ = 0.08, *OP* = 0.93) (Figure 1). However, there was no significant effect of sex (*p* = 0.115) on testosterone concentration. Moreover, age (*p* = 0.332), BMI (*p* = 0.511), and puberty (*p* = 0.679) did not influence testosterone concentration. Thus, the analyses were reported without any covariate. 

There was a significant effect of group (*p* = 0.008), but there was no effect of time (*p* = 0.107) nor time–group interaction (*p* = 0.077). The repeated-measures ANOVA results are reported in Table 4. 

### 3.6. Relationship between Testosterone and Fine Motor Skills

According to the Spearman’s correlation coefficient, there was no relevant relationship (*p* > 0.050) between the changes in testosterone concentration and altered fine motor skills in any of the groups (Table 5). However, we found a significant relationship (*p* = 0.034, *r* = 0.31) between the post-test concentration of testosterone and the post-test score of fine motor skills among participants in the CVE group (Table 5).

## 4. Discussion

Our findings indicate that fine motor skills and testosterone concentration did not change after either the CE or CVE training. Moreover, we failed to find a significant relationship between changes in testosterone concentration and altered fine motor skills. However, there was a positive correlation between the post-test concentration of testosterone and the post-test score of fine motor skills following the CVE training.

As mentioned in the Methods section, we measured cardiorespiratory fitness and gross motor performance pre and post as a marker that the exercise interventions (CVE and CE accordingly) were sufficient and effective to make changes in the outcomes (fine motor skills and testosterone concentration). The results of the shuttle run test confirmed the effectiveness of CVE (participants in the CVE group improved their cardiorespiratory fitness) while participants in the CE group showed a significant drop in cardiorespiratory fitness. We confirmed the results of previous studies showing an improvement in shuttle run test after the CVE [6,52]. 

Regarding the gross motor skills [53], we used two tests: the flamingo balance test and the hand–eye coordination test. Static balance performance was improved only in the CE condition while the improvement of hand–eye coordination was seen in both CE and CVE groups. CE improved static balance and hand–eye coordination since CE is based on motor learning [21]. However, the CVE training induced an improvement in both hand–eye coordination and cardiorespiratory fitness, although the intensity of CVE and CE was the same. As mentioned in the Introduction section, CVE not only induces cardiovascular changes, but also stimulates brain areas responsible for motor control [16]. Thus, we prefer the CVE program for training sessions in adolescents at school. 

### 4.1. Effect of CE and CVE Training on Fine Motor Skills 

There were no significant changes in fine motor skills in any of the two experimental groups, but a significant deterioration in fine motor skills was seen in the CON group. However, a descriptive analysis showed that participants in the CVE made fewer mistakes after the intervention. Torabi et al. [54] found that long-term, high-intensity interval training (based on running) improved fine and gross motor skills in adolescents with attention deficit hyperactivity disorder. On the other hand, Brown [55] found an improvement in fine motor skills after a long-term intervention with motor activities in young children. Analyzing the acute studies, Budde et al. [56] concluded that CE positively affected attention in adolescents, which is important for fine motor skills [57]. However, the findings of our study showed the opposite—participants in the CE training group did not demonstrate significant improvement in fine motor skills. Moreover, Wegner et al. [23] reported that acute psychosocial stress and not a bout of moderate intensity exercise affected fine motor skills in adolescents. Thus, we failed to confirm our expectation that CE training would be beneficial for improving fine motor skills.

### 4.2. Effect of CE and CVE Training on Testosterone 

There were no significant changes in testosterone concentration after the intervention and the effect of time–group interaction was minimal (*η*^2^ = 0.041). Our results are consistent with the study carried out by Akko et al. [26] where the authors did not find changes in salivary testosterone after the 10 weeks of cardiovascular vs. motor exercise training of moderate intensity in preadolescent children. Indeed, most studies have analyzed testosterone concentration in response to an acute intervention and found increased testosterone concentration after the exercise of moderate–vigorous intensity in adolescents [58,59]. Moreover, Wilkerson et al. [60] found an increased plasma testosterone after an acute intervention of light intensity CVE in young adults. These different findings may be explained in part by the exercise intensity, but an acute exercise and exercise training are completely different interventions and the application of each should be analyzed separately [14,15].

### 4.3. Association between Testosterone and Fine Motor Skills 

A medium positive correlation between the post-test testosterone concentration and post-test fine motor skills score was found only in the CVE group. However, we did not expect the relationship meaning a detrimental effect of the testosterone on fine motor skills: participants with a higher testosterone concentration made more mistakes in the fine motor skills test. On the contrary, Akko et al. [26] revealed the positive impact of increased testosterone on cognition after the motor exercise training in preadolescent children. Moreover, a study by Wegner et al. [23] found an association between the overall increased salivary testosterone and the overall improvement of fine motor skills in adolescents, although after an acute stress. Acute exercise is supposed to induce immediate chemical changes while an exercise training is expected to affect morphological brain changes [61]. An animal study showed an increased capillary density within three days of the onset of running activity in mice [62]. Thus, an accumulating effect of every single exercise session on the brain might be expected after the longitudinal intervention. It could be speculated that a bout of CVE activated a release of testosterone [59], which influenced the brain [30] and in the long-term period–fine motor skills. Fine motor skills tend to be controlled by the cerebellum [63,64], which is affected by testosterone [65]. While the exercise intensity was the same in both experimental groups in our study, the type of exercise seems to be an important characteristics of exercise training intervention in the analysis of fine motor skills and testosterone. It seems that CVE tended to induce a detrimental effect of testosterone on fine motor skills. To our knowledge, this is the first longitudinal study analyzing the relationship between fine motor skills and salivary testosterone after CVE vs. CE training in adolescents. 

### 4.4. Limitations and Directions for Further Research 

Among the limitations of this research, it is important to note that we have no follow-up data to demonstrate any lasting effects of CVE and CE training over longer periods after the intervention. Regarding the relationship between fine motor skills and salivary testosterone, the results should be interpreted with caution due to the magnitude of correlation observed (*r* = 0.31). While the drawing trail test requires attention and accuracy, future studies should include a parallel evaluation of cognitive functions. Furthermore, we used only one test to evaluate fine motor skills. Other methods, such as Functional dexterity test [66], might be used together with the drawing trail test for further research and extend the results of fine motor skills after the exercise training. Though shuttle run and gross motor tests showed the effectiveness of CE and CVE training programs, it was not enough to get a significant change in the performance of fine motor skills. Thus, subjective evaluation of training load would be useful to monitor the exercise program [15]. Finally, the samples were not measured in duplicates and were collected just once before and once after the intervention. 

## 5. Conclusions

Our findings demonstrated that neither CE nor CVE training was significantly effective to improve fine motor skills and change the testosterone concentration in adolescents. Nevertheless, post-test salivary testosterone concentration was positively correlated to post-test fine motor skills among those participants who underwent the CVE training. These results might be of interest to distinguish what exercise program may be the best for specific outcomes when incorporated into in-school physical education or after-school activities for adolescents. CVE training showed an effect on the relationship between fine motor skills and salivary testosterone together with the improvement in hand–eye coordination and cardiorespiratory fitness. Thus, researchers should focus on this type of exercise training.

## Figures and Tables

**Table 1:** Mean values (SD) for the basic characteristics of the two experimental groups (CE and CVE) and the control group (CON).

Characteristic	CON	CE	CVE
Age (years)	12.43 (0.62)	13.15 (0.99)	12.73 (0.79)
BMI (kg/m^2^)	19.22 (2.12)	19.41 (1.95)	19.54 (2.46)
Tanner (stage)	3.37 (0.68)	3.29 (0.93)	3.10 (0.66)

Note. CON = control group; CE = coordinative exercise; CVE = cardiovascular exercise; BMI = body mass index.

**Table 2:** Example of intervention exercises.

Exercise	Aim	Tasks	Modification
Cardiovascular exercise (game)	To catch a ball faster than your competitor	Participants are divided in two groups and successively calculated. Physical Education teacher throws a ball and calls a random number. Those participants (according to the calculation) must run and catch a ball.	Fast walking instead of running; Running sideways
		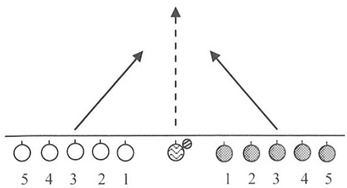	
Coordinative exercise (game)	To complete the task 10 times and do it faster than your competitor	Participants break into pairs. One of the participants jumps over a jumping rope and throws a rope on the ground that forms a loop. The participant stands on one leg in the loop and says “one”. After that, the participant jumps over a rope twice and stands in the loop while says “one, two”, etc. The participant repeats the task until makes a mistake (e.g., losing balance, not forming a loop) and gives the rope to the competitor.	Standing on one leg and clapping hands; Saying letters instead of numbers
		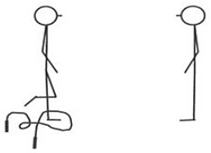	

**Table 3:** Median values (25th and 75th percentiles) for cardiovascular endurance, gross motor and fine motor skills in the two experimental (CE and CVE) groups and the control group (CON).

Variable	CON	CE	CVE
Shuttle run test (score)			
pre	4.0 (3.0, 5.0)	4.0 (3.0, 5.3)	4.0 (3.0, 4.5)
post	5.0 (4.0, 6.0) ***	3.0 (2.0, 5.0) ***	6.0 (4.0, 7.0) ***
Flamingo balance test (score)			
pre	7.0 (3.0, 15.0)	7.0 (5.0, 11.0)	8.0 (7.0, 12.3)
post	12.0 (5.0, 18.0) **	4.0 (3.0, 10.0) **	10.0 (7.0, 13.0)
Hand–eye coordination test (score)			
pre	14.0 (11.0, 16.0)	15.0 (13.0, 20.0)	15.0 (12.0, 18.0)
post	13.0 (10.0, 18.0)	18.0 (16.0, 23.0) ***	19.0 (17.0, 22.0) ***
Drawing trail test (score)			
pre	1.0 (0.0, 3.0)	2.0 (0.0, 3.0)	2.0 (1.0, 3.0)
post	2.0 (1.0, 4.0) *	2.0 (1.0, 4.0)	1.0 (0.0, 4.0)

Note. CON = control group. CE = coordinative exercise. CVE = cardiovascular exercise. * *p* < 0.050, ** *p* < 0.010, and *** *p* < 0.001 indicate a difference with pre-test values within the group.

**Table 4:** Repeated-measures ANOVA results for testosterone concentration.

Factor	df	F	Sig.	Partial Eta Squared	Observed Power
Time (within-subject)	1	2.638	0.107	0.021	0.364
Group (between-trial)	2	4.986	0.008	0.074	0.804
Time × Group	2	2.620	0.077	0.041	0.513

**Table 5:** Spearman’s correlation coefficients for pre-, post-test, difference values (delta) of testosterone concentration, and fine motor skills in the two experimental groups (CE and CVE) and the control group (CON).

Variable	CON			CE			CVE		
	T(Pre)	T(Post)	ΔT	T(Pre)	T(Post)	ΔT	T(Pre)	T(Post)	ΔT
T(pre)	—			—			—		
T(post)	0.30	—		0.74 ***	—		0.57 ***	—	
ΔT	−0.23	0.51 ***	—	−0.39 *	0.26	—	−0.14	0.45 **	—
DTT(pre)	0.02	0.23	0.20	−0.10	0.04	0.21	0.03	0.07	0.18
DTT(post)	0.17	0.14	0.03	−0.03	−0.05	−0.03	0.14	0.31 *	0.18
ΔDTT	0.24	0.01	−0.13	0.07	−0.09	−0.24	0.05	0.18	−0.06

Note. CON = control group. CE = coordinative exercise. CVE = cardiovascular exercise. T = Testosterone. DTT = Drawing trail test. * *p* < 0.050; ** *p* < 0.010; *** *p* < 0.001.

## Data Availability

Data sharing is not applicable to this article.

## References

[1] Biddle S.J., Ciaccioni S., Thomas G., Vergeer I. (2019). Physical activity and mental health in children and adolescents: An updated review of reviews and an analysis of causality. Psychol. Sport Exerc..

[2] Boreham C., Riddoch C. (2001). The physical activity, fitness and health of children. J. Sports Sci..

[3] Janssen I., LeBlanc A.G. (2010). Systematic review of the health benefits of physical activity and fitness in school-aged children and youth. Int. J. Behav. Nutr. Phys. Act..

[4] Diamond A. (2000). Close interrelation of motor development and cognitive development and of the cerebellum and prefrontal cortex. Child Dev..

[5] Young C.J., Levine S.C., Mix K.S. (2018). The connection between spatial and mathematical ability across development. Front. Psychol..

[6] Koutsandréou F., Wegner M., Niemann C., Budde H. (2016). Effects of motor versus cardiovascular exercise training on children’s working memory. Med. Sci. Sports Exerc..

[7] Grissmer D., Grimm K.J., Aiyer S.M., Murrah W.M., Steele J.S. (2010). Fine motor skills and early comprehension of the world: Two new school readiness indicators. Dev. Psychol..

[8] Gaul D., Issartel J. (2016). Fine motor skill proficiency in typically developing children: On or off the maturation track?. Hum. Mov. Sci..

[9] Geertsen S.S., Thomas R., Larsen M.N., Dahn I.M., Andersen J.N., Krause-Jensen M., Korup V., Nielsen C.M., Wienecke J., Ritz C. (2016). Motor skills and exercise capacity are associated with objective measures of cognitive functions and academic performance in preadolescent children. PLoS ONE.

[10] Gentier I., D’Hondt E., Shultz S., Deforche B., Augustijn M., Hoorne S., Verlaecke K., de Bourdeaudhuij I., Lenoir M. (2013). Fine and gross motor skills differ between healthy-weight and obese children. Res. Dev. Disabil..

[11] Payne V.G., Isaacs L.D., Payne V.G., Isaacs L.D. (2017). Fine motor development. Human Motor Development: A Lifespan Approach.

[12] Pozo-Rico T., Sandoval I. (2020). Can Academic Achievement in Primary School Students Be Improved Through Teacher Training on Emotional Intelligence as a Key Academic Competency?. Front. Psychol..

[13] Steinmayr R., Weidinger A.F., Schwinger M., Spinath B. (2019). The importance of students’ motivation for their academic achievement–replicating and extending previous findings. Front. Psychol..

[14] Budde H., Schwarzc R., Velasques B., Ribeiro P., Holzweg M., Machado S., Brazaitis M., Staack F., Wegner M. (2016). The need for differentiating between exercise, physical activity, and training. Autoimmun. Rev..

[15] Gronwald T., Törpel A., Herold F., Budde H. (2020). Perspective of Dose and Response for Individualized Physical Exercise and Training Prescription. J. Funct. Morphol. Kinesiol..

[16] Wagner G., Herbsleb M., de la Cruz F., Schumann A., Köhler S., Puta C., Gabriel H.W., Reichenbach J.R., Bär K.J. (2017). Changes in fMRI activation in anterior hippocampus and motor cortex during memory retrieval after an intense exercise intervention. Biol. Psychol..

[17] Riebe D., Pescatello L.S., Arena R., Riebe D., Thompson P.D. (2014). General Principles of exercise Prescription. ACSM’s Guidelines for Exercise Testing and Prescription.

[18] Voelcker-Rehage C., Godde B., Staudinger U.M. (2011). Cardiovascular and coordination training differentially improve cognitive performance and neural processing in older adults. Front. Hum. Neurosci..

[19] Spampinato D.A., Celnik P.A., Rothwell J.C. (2020). Cerebellar–Motor Cortex Connectivity: One or Two Different Networks?. J. Neurosci..

[20] Stoodley C.J., Valera E.M., Schmahmann J.D. (2012). Functional topography of the cerebellum for motor and cognitive tasks: An fMRI study. Neuroimage.

[21] Voelcker-Rehage C., Niemann C. (2013). Structural and functional brain changes related to different types of physical activity across the life span. Neurosci. Biobehav. Rev..

[22] Black J.E., Isaacs K.R., Anderson B.J., Alcantara A.A., Greenough W.T. (1990). Learning causes synaptogenesis, whereas motor activity causes angiogenesis, in cerebellar cortex of adult rats. Proc. Natl. Acad. Sci. USA.

[23] Wegner M., Koedijker J.M., Budde H. (2014). The effect of acute exercise and psychosocial stress on fine motor skills and testosterone concentration in the saliva of high school students. PLoS ONE.

[24] Grandys M., Majerczak J., Duda K., Zapart-Bukowska J., Kulpa J., Zoladz J.A. (2009). Endurance training of moderate intensity increases testosterone concentration in young, healthy men. Int. J. Sports Med..

[25] Hayes L.D., Herbert P., Sculthorpe N.F., Grace F.M. (2017). Exercise training improves free testosterone in lifelong sedentary aging men. Endocr. Connect..

[26] Akko D.P., Koutsandreou F., Murillo-Rodriguez E., Wegner M., Budde H. (2020). The effects of an exercise training on steroid hormones in preadolescent children-A moderator for enhanced cognition?. Physiol. Behav..

[27] Niemann C., Wegner M., Voelcker-Rehage C., Holzweg M., Arafat A.M., Budde H. (2013). Influence of acute and chronic physical activity on cognitive performance and saliva testosterone in preadolescent school children. Ment. Health Phys. Act..

[28] Fargo K.N., Foecking E.M., Jones K.J., Sengelaub D.R. (2009). Neuroprotective actions of androgens on motoneurons. Front. Neuroendocrinol..

[29] Byers J.S., Huguenard A.L., Kuruppu D., Liu N.K., Xu X.M., Sengelaub D.R. (2012). Neuroprotective effects of testosterone on motoneuron and muscle morphology following spinal cord injury. J. Comp. Neurol..

[30] Oki K., Law T.D., Loucks A.B., Clark B.C. (2015). The effects of testosterone and insulin-like growth factor 1 on motor system form and function. Exp. Gerontol..

[31] Tammelin T., Ekelund U., Remes J., Näyhä S. (2007). Physical activity and sedentary behaviors among Finnish youth. Med. Sci. Sports Exerc..

[32] WHO Multicentre Growth Reference Study Group (2006). Reliability of anthropometric measurements in the WHO Multicentre Growth Reference Study. Acta Paediatr. Suppl..

[33] Vasold K.L., Parks A.C., Phelan D., Pontifex M.B., Pivarnik J.M. (2019). Reliability and Validity of Commercially Available Low-Cost Bioelectrical Impedance Analysis. Int. J. Sport Nutr. Exerc. Metab..

[34] Rasmussen A.R., Wohlfahrt-Veje C., de Renzy-Martin K.T., Hagen C.P., Tinggaard J., Mouritsen A., Mieritz M.G., Main K.M. (2015). Validity of self-assessment of pubertal maturation. Pediatrics.

[35] Chavarro J.E., Watkins D.J., Afeiche M.C., Zhang Z., Sánchez B.N., Cantonwine D., Mercado-García A., Blank-Goldenberg C., Meeker J.D., Téllez-Rojo M.M. (2017). Validity of Self-Assessed Sexual Maturation Against Physician Assessments and Hormone Levels. J. Pediatr..

[36] Goodie J.L., Larkin K.T., Schauss S. (2000). Validation of Polar heart rate monitor for assessing heart rate during physical and mental stress. J. Psychophysiol..

[37] Freedson P.S., Cureton K.J., Heath G.W. (2000). Status of field-based fitness testing in children and youth. Prev. Med..

[38] Mayorga-Vega D., Aguilar-Soto P., Viciana J. (2015). Criterion-Related Validity of the 20-M Shuttle Run Test for Estimating Cardiorespiratory Fitness: A Meta-Analysis. J. Sports Sci. Med..

[39] Kemper H.C., Van Mechelen W. (1996). Physical Fitness Testing of Children: A European Perspective. Pediatr. Exerc. Sci..

[40] Sember V., Grošelj J., Pajek M. (2020). Balance Tests in Pre-Adolescent Children: Retest Reliability, Construct Validity, and Relative Ability. Int. J. Environ. Res. Public Health.

[41] Beashel P., Taylor J., Beashel P., Taylor J. (1997). Fitness For Health And Performance. The World of Sport Examined.

[42] Faber I.R., Oosterveld F.G., Nijhuis-Van der Sanden M.W. (2014). Does an eye-hand coordination test have added value as part of talent identification in table tennis? A validity and reproducibility study. PLoS ONE.

[43] Petermann F., Bös K., Kastner J. (2010). Movement Assessment Battery for Children-2 (Movement ABC-2): Deutsprachige Adaptation. Manual.

[44] Gísladóttir T., Haga M., Sigmundsson H. (2019). Motor Competence in Adolescents: Exploring Association with Physical Fitness. Sports.

[45] Crewther B.T., Lowe T.E., Ingram J., Weatherby R.P. (2010). Validating the salivary testosterone and cortisol concentration measures in response to short high-intensity exercise. J. Sports Med. Phys. Fitness..

[46] Vilūnienė A., Trinkūnienė L. (2014). Šiuolaikinė kūno kultūros pamoka: žaidimai. Modern Physical Education Lesson: Games.

[47] Machado F.A., Denadai B.S. (2011). Validity of maximum heart rate prediction equations for children and adolescents. Arq. Bras. Cardiol..

[48] Garber C.E., Blissmer B., Deschenes M.R., Franklin B.A., Lamonte M.J., Lee I., Nieman D.C., Swain D.P. (2011). American college of sports medicine position stand. quantity and quality of exercise for developing and maintaining cardiorespiratory, musculoskeletal, and neuromotor fitness in apparently healthy adults: Guidance for prescribing exercise. Med. Sci. Sports Exerc..

[49] Ferguson C. (2009). An Effect Size Primer: A Guide for Clinicians and Researchers. Prof. Psychol. Res. Pr..

[50] Fritz C.O., Morris P.E., Richler J.J. (2012). Effect size estimates: Current use, calculations, and interpretation. J. Exp. Psychol. Gen..

[51] Cohen J. (1988). Statistical Power Analysis for the Behavioral Sciences.

[52] Litleskare S., Enoksen E., Sandvei M., Støen L., Stensrud T., Johansen E., Jensen J. (2020). Sprint Interval Running and Continuous Running Produce Training Specific Adaptations, Despite a Similar Improvement of Aerobic Endurance Capacity—A Randomized Trial of Healthy Adults. Int. J. Environ. Res. Public Health.

[53] Lubans D.R., Morgan P.J., Cliff D.P., Barnett L.M., Okely A.D. (2010). Fundamental Movement Skills in Children and Adolescents. Sports Med..

[54] Torabi F., Farahani A., Safakish S., Ramezankhani A., Dehghan F. (2018). Evaluation of motor proficiency and adiponectin in adolescent students with attention deficit hyperactivity disorder after high-intensity intermittent training. Psychiatry Res..

[55] Brown C.G. (2010). Improving fine motor skills in young children: An intervention study. Educ. Psychol. Pract..

[56] Budde H., Voelcker-Rehage C., Pietrassyk-Kendziorra S., Ribeiro P., Tidow G. (2008). Acute coordinative exercise improves attentional performance in adolescents. Neurosci. Lett..

[57] Rinne P., Hassan M., Fernandes C., Han E., Hennessy E., Waldman A., Sharma P., Soto D., Leech R., Malhotra P.A. (2018). Motor dexterity and strength depend upon integrity of the attention-control system. Proc. Natl. Acad. Sci. USA.

[58] Budde H., Pietrassyk-Kendziorra S., Bohm S., Voelcker-Rehage C. (2010). Hormonal responses to physical and cognitive stress in a school setting. Neurosci. Lett..

[59] Budde H., Voelcker-Rehage C., Pietrassyk-Kendziorra S., Machado S., Ribeiro P., Arafat A.M. (2010). Steroid hormones in the saliva of adolescents after different exercise intensities and their influence on working memory in a school setting. Psychoneuroendocrinology.

[60] Wilkerson J.E., Horvath S.M., Gutin B. (1980). Plasma testosterone during treadmill exercise. J. Appl. Physiol..

[61] Best J.R. (2010). Effects of physical activity on children’s executive function: Contributions of experimental research on aerobic exercise. Dev. Rev..

[62] Van der Borght K., Kóbor-Nyakas D.É., Klauke K., Eggen B.J., Nyakas C., Van der Zee E., Meerlo P. (2009). Physical exercise leads to rapid adaptations in hippocampal vasculature: Temporal dynamics and relationship to cell proliferation and neurogenesis. Hippocampus.

[63] Miall R.C., Reckess G.Z., Imamizu H. (2001). The cerebellum coordinates eye and hand tracking movements. Nat. Neurosci..

[64] Thomas A.R., Lacadie C., Vohr B., Ment L.R., Scheinost D. (2017). Fine motor skill mediates visual memory ability with microstructural neuro-correlates in cerebellar peduncles in prematurely born adolescents. Cereb. Cortex.

[65] Schutter D.J., Meuwese R., Bos M.G., Crone E.A., Peper J.S. (2017). Exploring the role of testosterone in the cerebellum link to neuroticism: From adolescence to early adulthood. Psychoneuroendocrinology.

[66] Duff S.V., Aaron D.H., Gogola G.R., Valero-Cuevas F.J. (2015). Innovative evaluation of dexterity in pediatrics. J. Hand Ther..

